# Age- and Treatment-Specific TP53 and PI3K Alterations in Pancreatic Ductal Adenocarcinoma (PDAC) Revealed by Conversational Artificial Intelligence

**DOI:** 10.3390/ijms27114981

**Published:** 2026-05-30

**Authors:** Fernando C. Diaz, Brigette Waldrup, Francisco G. Carranza, Sophia Manjarrez, Enrique Velazquez-Villarreal

**Affiliations:** 1 Lineberger Comprehensive Cancer Center, University of North Carolina, Chapel Hill, NC 27514, USA; 2 Department of Integrative Translational Sciences, Duarte, CA 91010, USA; 3 City of Hope, Comprehensive Cancer Center, Duarte, CA 91010, USA

**Keywords:** pancreatic ductal adenocarcinoma, precision oncology, gemcitabine, artificial intelligence, LLM, conversational AI, AI-agents, TP53 pathway, PI3K pathway

## Abstract

Despite its marked molecular heterogeneity and variable response to gemcitabine-based therapies, pancreatic ductal adenocarcinoma (PDAC) remains poorly understood at the pathway level, particularly across age and treatment contexts. We applied a conversational artificial intelligence framework (AI-HOPE-TP53 and AI-HOPE-PI3K) to analyze clinical and genomic data from 184 PDAC tumors stratified by age and gemcitabine exposure. Pathway-centric analyses were performed and validated using conventional statistical methods. TP53 alterations were more frequent in early-onset compared to late-onset PDAC among gemcitabine-treated patients and showed a similar trend in early-onset untreated cases. In late-onset PDAC without gemcitabine exposure, absence of TP53 alterations was associated with improved overall survival. PI3K alterations were enriched in late-onset gemcitabine-treated tumors. Notably, late-onset patients without PI3K alterations who were not treated with gemcitabine demonstrated significantly improved survival. TP53 and PI3K pathway dependencies in PDAC are context-specific, varying by age and treatment exposure. These findings highlight the value of conversational AI for integrative precision oncology analyses and molecular stratification.

## 1. Introduction

Late-stage diagnosis, profound molecular heterogeneity, and a highly immunosuppressive tumor microenvironment define pancreatic ductal adenocarcinoma (PDAC), contributing to its status as one of the most aggressive and therapeutically refractory malignancies [1,2,3]. While gemcitabine-based regimens remain central to clinical management, their efficacy is limited by rapid onset of chemoresistance and context-dependent variability in outcomes [4,5,6]. This resistance is mediated not only by tumor-intrinsic genetic alterations but also by intricate tumor–stroma interactions, including those involving cancer-associated fibroblasts (CAFs), immune cells, and extracellular matrix remodeling, which collectively regulate tumor evolution and treatment response [7,8,9].

Among the key molecular determinants of PDAC biology, alterations in tumor suppressor pathways and survival signaling networks are particularly critical. Mutations in TP53 occur in the majority of PDAC cases and are associated with genomic instability, impaired apoptosis, and enhanced tumor progression [1,10]. Beyond its canonical tumor suppressor role, TP53 dysfunction has been increasingly linked to immune evasion mechanisms and remodeling of the tumor microenvironment, further contributing to poor clinical outcomes [11]. In parallel, aberrant activation of the PI3K/AKT signaling pathway plays a central role in promoting tumor cell survival, metabolic adaptation, and resistance to cytotoxic therapies, including gemcitabine [4,12,13]. Activation of this pathway has been shown to regulate key processes such as glycolysis, epithelial, mesenchymal transition (EMT), and drug efflux, thereby sustaining tumor aggressiveness and therapeutic escape [14,15].

Importantly, PI3K pathway dysregulation is not a static feature but evolves dynamically under therapeutic pressure. Multiple studies have demonstrated that inhibition of PI3K/AKT signaling can enhance gemcitabine sensitivity, while its activation contributes to resistance through mechanisms including metabolic reprogramming, anti-apoptotic signaling, and modulation of drug transport systems [4,16,17,18]. Similarly, emerging evidence suggests that TP53 status may influence treatment response and disease progression in a context-dependent manner, highlighting the need to move beyond single-gene paradigms toward integrative, pathway-level analyses that reflect the complexity of PDAC biology [10,11].

These molecular processes are further modulated by the tumor microenvironment, where cytokine signaling and stromal interactions reinforce resistance phenotypes. For example, TGF-β-mediated signaling contributes to fibroblast activation, immune suppression, and chemoresistance, while cross-talk with pathways such as JAK/STAT and PI3K amplifies tumor-promoting signals [7,8,19]. Additionally, chemotherapy itself can induce adaptive responses, including immune checkpoint upregulation and metabolic shifts, which may paradoxically support tumor persistence despite initial cytotoxic effects [20,21,22]. Collectively, these findings underscore the necessity of understanding how key oncogenic and tumor suppressor pathways interact within clinically relevant contexts, including patient age and treatment exposure.

A critical challenge in precision oncology is the ability to systematically interrogate these complex interactions across large, heterogeneous clinical–genomic datasets [23,24,25,26,27,28,29,30]. Traditional analytical frameworks often lack the flexibility to rapidly construct and refine multi-dimensional cohorts stratified by clinically meaningful variables such as age of onset and therapeutic exposure. This limitation is particularly relevant in PDAC, where early-onset and late-onset disease may represent biologically distinct entities with differential pathway dependencies and clinical trajectories [31,32].

We applied a conversational artificial intelligence framework to address this gap by enabling dynamic cohort construction and pathway-focused analysis of TP53 and PI3K alterations in PDAC. Building on prior AI-driven pathway interrogation approaches, AI-HOPE-TP53 [33] and AI-HOPE-PI3K [34], this method allows rapid hypothesis generation and testing across clinically stratified groups. We evaluated the distribution and clinical relevance of these alterations across age- and treatment-defined subgroups and assessed their associations with survival outcomes. These analyses aimed to uncover context-specific molecular dependencies that could guide precision oncology strategies, including combination therapies to overcome chemoresistance.

## 2. Results

### 2.1. Clinical Profile of the PDAC Cohort

As shown in Table 1, the study cohort comprised 184 patients with PDAC, each with comprehensive demographic, clinical, and molecular annotations. All molecular analyses were derived from primary tumor specimens, ensuring consistency and comparability across the dataset.

The cohort was predominantly composed of patients with late-onset PDAC (≥50 years), with gemcitabine-treated individuals representing the largest subgroup, followed by untreated cases. Early-onset PDAC (<50 years) constituted a smaller fraction of the cohort. This distribution reflects both the higher prevalence of late-onset disease and the central role of gemcitabine-based regimens in clinical practice.

Sex distribution was relatively balanced, with a slight predominance of male patients. All samples were obtained from primary tumors, minimizing potential confounding from metastatic heterogeneity or treatment-induced clonal evolution.

Disease stage at diagnosis was primarily stage II, with fewer cases in early-stage disease (stage I) and limited representation of advanced stages (stage III and IV). A small subset of patients had unavailable staging information.

Most patients were classified as not Hispanic or Latino, with a smaller proportion identified as Hispanic or Latino and a notable fraction with unknown ethnicity.

Together, these baseline characteristics define a clinically representative PDAC cohort and provide the foundation for subsequent analyses of age- and treatment-specific patterns of TP53 and PI3K pathway alterations and their association with clinical outcomes.

### 2.2. Patterns of TP53 and PI3K Pathway Alterations Across Age and Treatment Contexts

Stratified analysis by age and gemcitabine exposure (Table 2a–d) revealed divergent patterns between TP53 and PI3K pathways. TP53 alterations were pervasive across all subgroups, while PI3K alterations demonstrated pronounced context-dependent variability, most notably in late-onset PDAC.

#### 2.2.1. TP53 Pathway Alterations Across Age and Treatment Strata

TP53 pathway alterations were highly prevalent across the cohort, consistent with their central role in PDAC biology. Within early-onset PDAC, TP53 alterations were observed in 80.0% of gemcitabine-treated tumors compared to 40.0% in non-treated tumors, although this difference did not reach statistical significance (*p* = 0.1313). In late-onset PDAC, TP53 alterations were similarly frequent in both treatment groups, occurring in 62.6% of gemcitabine-treated and 64.4% of non-treated tumors (*p* = 0.9461) (Table 2a).

Comparisons across age groups within treatment strata revealed no statistically significant differences. Among gemcitabine-treated patients, TP53 alterations were present in 80.0% of early-onset tumors and 62.6% of late-onset tumors (*p* = 0.2496). In the non-treated setting, alteration frequencies were 40.0% in early-onset PDAC and 64.4% in late-onset PDAC (*p* = 0.3546) (Table 2b).

TP53 pathway disruption emerged as a pervasive feature of PDAC, remaining consistent across age groups and treatment contexts, with no statistically significant differences observed at the pathway level.

#### 2.2.2. PI3K Pathway Alterations Across Age and Treatment Strata

In contrast to TP53, PI3K pathway alterations were less frequent overall but demonstrated notable variability depending on clinical context. In early-onset PDAC, PI3K alterations were identified in 13.3% of gemcitabine-treated tumors and were absent in non-treated tumors (0.0%), although this difference was not statistically significant (*p* = 1).

In late-onset PDAC, a distinct pattern emerged. PI3K pathway alterations were detected in 13.2% of gemcitabine-treated tumors compared to only 2.7% of non-treated tumors, representing a statistically significant difference (*p* = 0.02266) (Table 2c). This finding suggests a potential association between PI3K pathway activation and gemcitabine exposure in late-onset disease.

Comparisons of PI3K alteration frequencies across age groups within treatment strata did not reveal significant differences. Among gemcitabine-treated patients, alteration rates were nearly identical between early-onset and late-onset PDAC, while in the non-treated group, PI3K alterations were absent in early-onset tumors and detected only at low frequency in late-onset cases (Table 2d).

Together, these results indicate divergent pathway behavior in PDAC. While TP53 alterations are broadly consistent across subgroups, PI3K alterations appear more context-dependent, with enrichment in gemcitabine-treated late-onset disease, suggesting a role in treatment-related molecular adaptation.

### 2.3. Gene-Level Landscape of TP53 and PI3K

Given the relative uniformity of TP53 pathway alterations and the context-dependent variability observed for PI3K signaling at the pathway level S, we next examined these pathways at single-gene resolution to uncover clinically relevant molecular patterns masked by aggregate analyses (Tables S1–S8). Across the cohort, TP53 emerged as the predominant alteration within the tumor suppressor axis, consistently detected across both early- and late-onset PDAC and irrespective of gemcitabine exposure, reinforcing its role as a foundational driver of genomic instability. In contrast, alterations within the PI3K pathway were less frequent but distributed across multiple genes, including catalytic and regulatory components, suggesting a more heterogeneous and context-dependent architecture of pathway activation. Importantly, discrete gene-level events within the PI3K signaling network appeared enriched in specific clinical subgroups, particularly in gemcitabine-exposed tumors, highlighting potential adaptive mechanisms linked to therapeutic pressure. These findings reveal that, while TP53 alterations define a ubiquitous backbone of PDAC biology, PI3K pathway dysregulation is shaped by both age and treatment context, uncovering molecular dependencies that are not evident at the pathway level alone.

#### 2.3.1. TP53 Gene-Level Landscape Reveals Age-Associated Enrichment in Gemcitabine-Treated Early-Onset PDAC

At the single-gene level, the TP53 pathway was dominated by alterations in TP53, with other pathway components occurring at low frequency across all subgroups. In early-onset PDAC, TP53 mutations were more frequently observed in gemcitabine-treated tumors (86.7%) compared to non-treated tumors (40.0%), representing a notable, although not statistically significant, difference (*p* = 0.0726) (Table S1). Other genes within the TP53 pathway, including MDM2, MDM4, CDKN1A, ATM, CHEK1/2, PTEN, and BBC3, were largely unaltered in this subgroup, with only sporadic events such as CDKN2A (13.3–20.0%) and ATR (6.7%) detected at low frequencies.

In contrast, late-onset PDAC demonstrated a more uniform distribution of TP53 mutations across treatment groups. TP53 alterations were present in 57.1% of gemcitabine-treated tumors and 60.3% of non-treated tumors (*p* = 0.8064), indicating no treatment-associated enrichment (Table S2). Similarly, other TP53 pathway genes exhibited low mutation frequencies without meaningful differences between treated and untreated cases. Notably, CDKN2A mutations were observed in approximately one-fifth of late-onset tumors (~19–20%), while alterations in genes such as ATM, ATR, and CHEK2 occurred infrequently.

When comparing age groups within the gemcitabine-treated cohort, a statistically significant difference emerged. TP53 mutations were significantly more prevalent in early-onset tumors (86.7%) compared to late-onset tumors (57.1%) (*p* = 0.0431) (Table S3), suggesting an age-dependent enrichment of TP53 alterations in the context of gemcitabine exposure. In contrast, no significant age-based differences were observed in the non-treated setting, where TP53 mutation frequencies were 40.0% in early-onset and 60.3% in late-onset PDAC (*p* = 0.396) (Table S4).

Overall, these findings indicate that while TP53 mutations represent a central and recurrent feature of PDAC, their distribution is not entirely uniform. Instead, a distinct enrichment of TP53 alterations is observed in gemcitabine-treated early-onset PDAC, whereas late-onset disease exhibits a more stable mutation pattern across treatment contexts. This suggests that TP53-driven tumor biology may be differentially shaped by age and therapeutic exposure, with potential implications for treatment response and disease progression.

#### 2.3.2. PI3K Gene-Level Landscape Suggests Treatment-Associated Diversification in Late-Onset PDAC

At the gene level, the PI3K pathway displayed a markedly lower overall mutation burden than the TP53 pathway, with alterations distributed across multiple low-frequency nodes rather than dominated by a single recurrent driver. In early-onset PDAC, PI3K-related alterations were rare and restricted to gemcitabine-treated tumors, where isolated mutations in PIK3CA and RPTOR were each detected in 6.7% of cases, while no alterations were observed in the non-gemcitabine-treated early-onset subgroup (Table S5). No mutations were identified in PTEN, PIK3R1, PIK3R2, PIK3R3, INPP4B, AKT1/2/3, PPP2R1A, TSC1, TSC2, STK11, RHEB, RICTOR, or MTOR in early-onset untreated tumors, underscoring the sparsity of PI3K pathway disruption in this setting.

A broader and more heterogeneous PI3K mutational spectrum was observed in late-onset PDAC, particularly among gemcitabine-treated patients. In this subgroup, PIK3CA mutations were the most frequent PI3K-axis event (4.4%), followed by lower-frequency alterations in PPP2R1A, TSC2, STK11, and RICTOR (each 2.2%), with additional sporadic events in PIK3R1, PIK3R2, PIK3R3, INPP4B, AKT2, AKT3, TSC1, MTOR, and RPTOR (Table S6). By contrast, non-treated late-onset tumors showed a substantially narrower landscape, with only STK11 mutations identified in 2.7% of cases and no detectable alterations in most other PI3K pathway genes (Table S6). Although individual gene-level comparisons did not reach statistical significance, the accumulation of multiple low-frequency events in treated late-onset tumors supports a treatment-contextual expansion of PI3K pathway complexity.

Age-based comparisons within the gemcitabine-treated cohort further emphasized this pattern. Early-onset treated tumors harbored only isolated PIK3CA and RPTOR events, whereas late-onset treated tumors exhibited a broader distribution of mutations involving upstream regulators, catalytic components, and mTOR-complex-related genes, including PIK3R1/2/3, INPP4B, AKT2/3, PPP2R1A, TSC1/2, STK11, RICTOR, and MTOR (Table S7). Despite this greater diversity in late-onset disease, no single gene differed significantly between age groups within the gemcitabine-treated stratum. Likewise, comparison of untreated early- versus late-onset PDAC revealed minimal PI3K pathway disruption overall, with only STK11 mutations detected in a small subset of late-onset untreated tumors (2.7%) and no alterations observed in early-onset untreated cases (Table S8).

Taken together, these findings indicate that PI3K pathway dysregulation in PDAC is not characterized by a single dominant gene-level event, but rather by low-frequency alterations across multiple signaling components. This pattern is most evident in gemcitabine-treated late-onset PDAC, where the pathway appears more genomically diversified than in early-onset or untreated disease, suggesting that therapeutic exposure may coincide with broader PI3K-axis molecular heterogeneity.

#### 2.3.3. Integrated Interpretation

Taken together, gene-level analyses reveal that the relative consistency observed at the pathway level conceals important age- and treatment-dependent differences in the underlying molecular architecture of PDAC. The TP53 axis is characterized by a dominant, recurrent alteration pattern driven primarily by TP53 itself, with a notable enrichment in gemcitabine-treated early-onset tumors, suggesting a potential interaction between tumor suppressor dysfunction and therapeutic exposure in this subgroup. In contrast, the PI3K pathway exhibits a fundamentally different structure, marked by low-frequency alterations distributed across multiple genes rather than a single dominant driver. This dispersed pattern is most evident in gemcitabine-treated late-onset PDAC, where an expanded spectrum of PI3K-related alterations, including components of upstream regulation, catalytic signaling, and mTOR complex activity, emerges. Conversely, early-onset and untreated tumors display minimal PI3K pathway disruption, indicating a more constrained signaling landscape in these contexts.

Overall, these findings highlight two distinct modes of pathway dysregulation in PDAC: a TP53-driven architecture that is broadly conserved but modulated by age and treatment, and a PI3K pathway that appears more plastic and context-dependent, with evidence of increased molecular diversification in association with gemcitabine exposure. Because many individual gene-level events occurred at low frequency, particularly within early-onset and PI3K-altered subgroups, these observations should be interpreted cautiously and viewed primarily as exploratory signals rather than definitive molecular associations.

### 2.4. Context-Specific Survival Impact of TP53 Pathway Alterations

**Figure 1:**
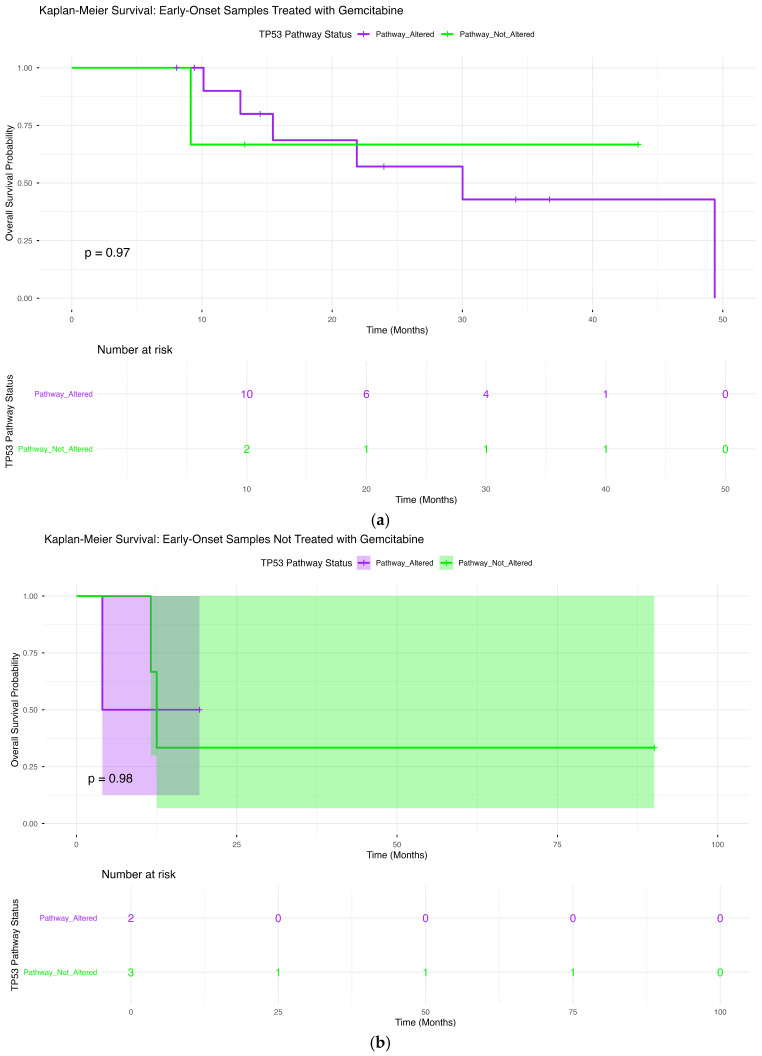

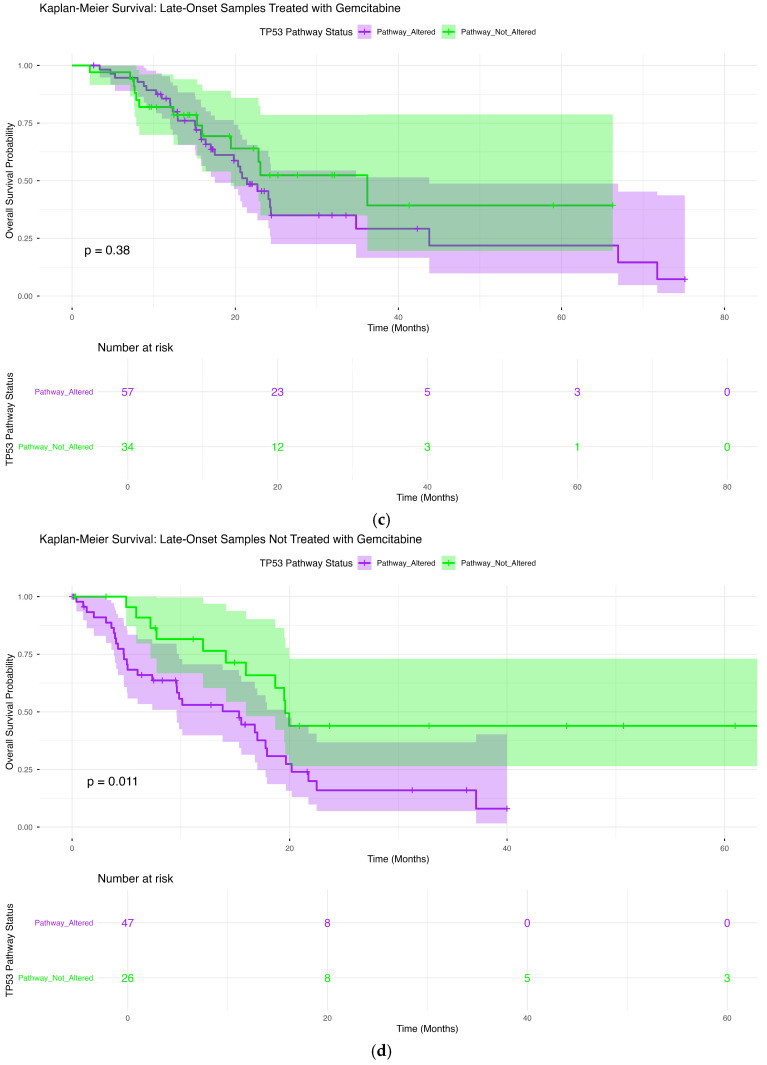
Overall survival stratified by TP53 pathway alteration status in age- and treatment-defined PDAC subgroups. Kaplan–Meier analyses illustrate differences in overall survival between patients with TP53 pathway-altered tumors and those lacking detectable TP53 alterations, with stratification based on age at diagnosis and gemcitabine exposure. The four panels represent: (**a**) early-onset PDAC (<50 years) receiving gemcitabine, (**b**) early-onset PDAC without gemcitabine treatment, (**c**) late-onset PDAC (≥50 years) receiving gemcitabine, and (**d**) late-onset PDAC without gemcitabine exposure. Confidence intervals (95%) are shown as shaded regions surrounding survival curves, and corresponding numbers at risk are provided below each panel.

To determine the prognostic implications of TP53 pathway dysregulation, we performed Kaplan–Meier survival analyses across PDAC subgroups stratified by age at diagnosis and gemcitabine exposure (Figure 1a–d).

#### 2.4.1. Early-Onset PDAC Under Gemcitabine Exposure

Among early-onset patients treated with gemcitabine (Figure 1a), overall survival was comparable between tumors harboring TP53 pathway alterations and those without detectable alterations (*p* = 0.97). Survival curves closely overlapped across the duration of follow-up, suggesting no discernible prognostic impact of TP53 status in this subgroup. The relatively small sample size contributed to broad confidence intervals, limiting sensitivity to detect subtle differences.

#### 2.4.2. Early-Onset PDAC Without Gemcitabine Exposure

In early-onset patients who did not receive gemcitabine (Figure 1b), TP53 pathway status similarly showed no association with overall survival (*p* = 0.98). Although slight variability in survival patterns was observed, no consistent separation between groups was evident. These findings should be interpreted cautiously given the limited number of patients and events.

#### 2.4.3. Late-Onset PDAC Under Gemcitabine Exposure

For late-onset PDAC patients undergoing gemcitabine-based therapy (Figure 1c), survival outcomes between TP53-altered and non-altered tumors demonstrated a modest degree of divergence; however, this did not achieve statistical significance (*p* = 0.38). While early survival was comparable, a gradual decline in survival probability was observed among TP53-altered cases at later time points. Nonetheless, overlapping confidence intervals indicate that these differences are not definitive.

#### 2.4.4. Late-Onset PDAC Without Gemcitabine Exposure

A distinct pattern was observed in late-onset patients not treated with gemcitabine (Figure 1d), where TP53 pathway status was significantly associated with survival outcomes (*p* = 0.011). Patients without TP53 alterations experienced improved overall survival compared to those with TP53 pathway mutations. The divergence between curves occurred early and persisted throughout follow-up, indicating a sustained adverse prognostic effect of TP53 alterations in this clinical context.

In summary, the impact of TP53 pathway alterations on survival in PDAC appears to depend on both age and treatment exposure. While no significant association was observed in early-onset disease or in gemcitabine-treated patients, TP53 alterations identify a subgroup with worse prognosis among late-onset, untreated patients. These findings should be interpreted cautiously given the limited subgroup sizes, particularly in early-onset PDAC, and should be considered exploratory and hypothesis-generating pending validation in larger independent cohorts.

### 2.5. Context-Specific Survival Impact of PI3K Pathway Alterations

**Figure 2:**
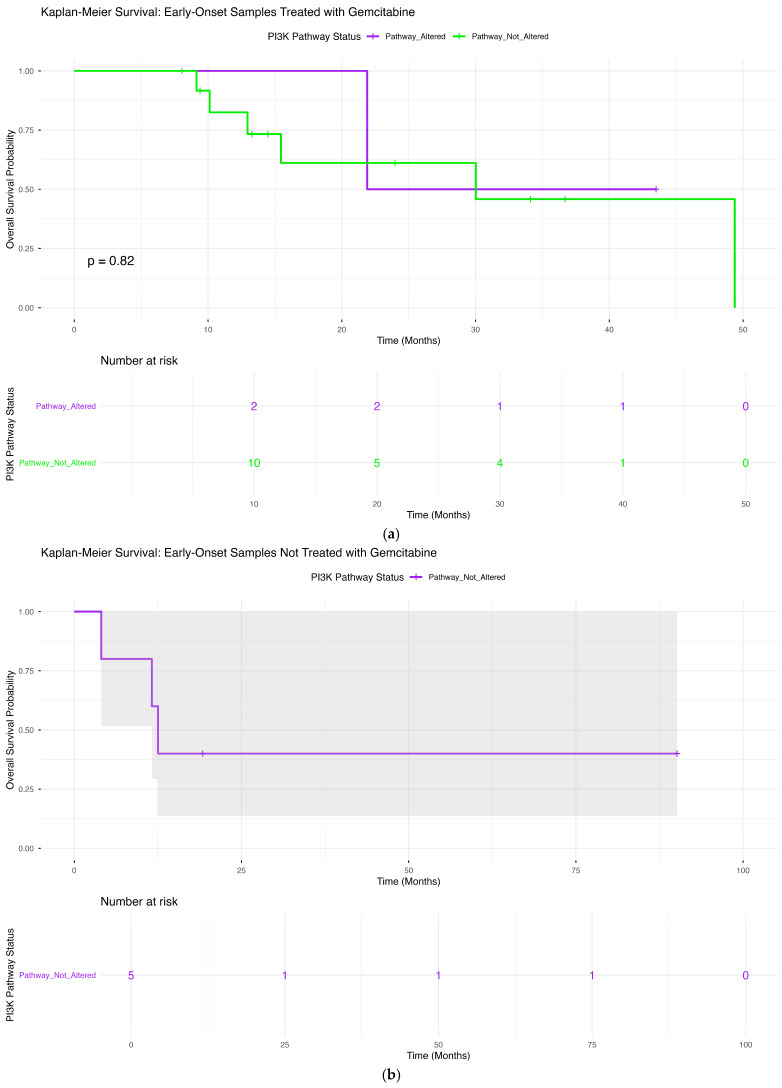

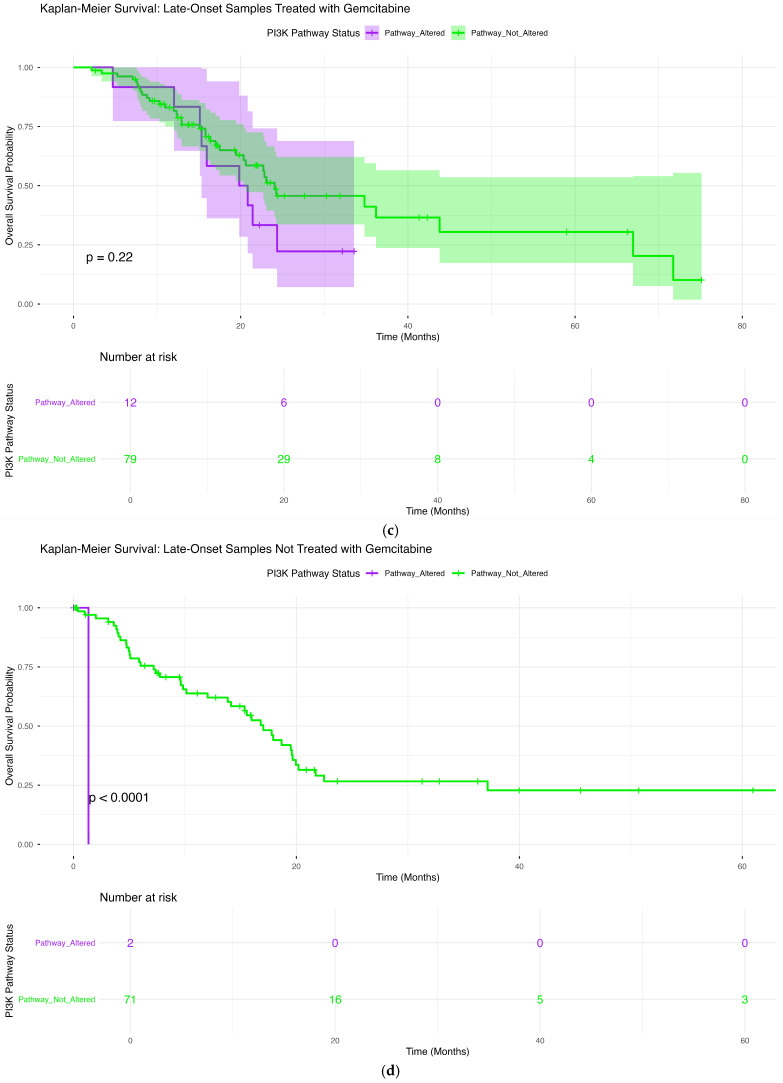
**Overall survival stratified by PI3K pathway alteration status in age- and treatment-defined PDAC subgroups.** Kaplan–Meier analyses compare survival outcomes between patients with PI3K pathway-altered tumors and those without detectable alterations, with stratification based on age at diagnosis and gemcitabine exposure. The panels represent: (**a**) early-onset PDAC (<50 years) receiving gemcitabine, (**b**) early-onset PDAC without gemcitabine exposure, (**c**) late-onset PDAC (≥50 years) receiving gemcitabine, and (**d**) late-onset PDAC without gemcitabine treatment. Within each subgroup, survival trajectories for PI3K-altered and PI3K-wild-type tumors are evaluated. Shaded regions denote 95% confidence intervals, and risk tables beneath each plot indicate the number of patients at risk over time, supporting interpretation of survival dynamics across follow-up.

We investigated the association between PI3K pathway alterations and overall survival across PDAC subgroups defined by age at diagnosis and gemcitabine exposure using Kaplan–Meier analyses (Figure 2a–d).

#### 2.5.1. Early-Onset PDAC with Gemcitabine Exposure

In early-onset patients receiving gemcitabine (Figure 2a), overall survival was comparable between PI3K-altered and PI3K-wild-type tumors (*p* = 0.82). The survival trajectories for both groups were largely overlapping throughout follow-up, with no consistent divergence observed. The relatively small size and imbalance of the PI3K-altered subgroup may increase susceptibility to unstable survival estimates and limit statistical robustness.

#### 2.5.2. Early-Onset PDAC Without Gemcitabine Exposure

For early-onset patients not treated with gemcitabine (Figure 2b), meaningful comparison by PI3K pathway status was not possible, as no PI3K alterations were detected in this subgroup. Consequently, survival estimates reflect only the PI3K-wild-type population, preventing assessment of differential outcomes.

#### 2.5.3. Late-Onset PDAC with Gemcitabine Exposure

Among late-onset patients treated with gemcitabine (Figure 2c), survival outcomes did not significantly differ according to PI3K pathway status (*p* = 0.22). Although PI3K-altered tumors showed a tendency toward lower survival probabilities at earlier time points, this pattern was not sustained, and the survival curves converged with substantial overlap in confidence intervals.

#### 2.5.4. Late-Onset PDAC Without Gemcitabine Exposure

In contrast, late-onset patients who did not receive gemcitabine (Figure 2d) exhibited a pronounced survival difference based on PI3K pathway status (*p* < 0.0001). Patients with PI3K pathway alterations had markedly reduced overall survival compared to those without alterations. The separation between survival curves occurred early and persisted over time, indicating a robust and sustained adverse prognostic effect.

Analysis revealed a context-dependent role of PI3K pathway alterations in PDAC survival. No significant effect was observed in early-onset or gemcitabine-treated patients, whereas PI3K alterations delineated a high-risk subgroup among untreated late-onset patients. These findings support the integration of pathway-level information with clinical variables to refine prognostic models and guide precision oncology.

### 2.6. AI-Enabled Identification of Context-Specific Molecular Patterns

#### 2.6.1. AI-HOPE-TP53: Context-Aware Cohort Modeling and Pathway Interrogation

To extend conventional pathway analyses, we utilized the AI-HOPE-TP53 framework to perform structured, natural language-driven exploratory analyses within the integrated PDAC clinical–genomic dataset. This approach enabled flexible cohort construction, real-time survival evaluation, and targeted enrichment testing under explicitly defined clinical and molecular conditions.

As an initial demonstration (Figure S1), the AI agent was tasked with constructing clinically matched cohorts based on age and treatment exposure and evaluating survival outcomes according to TP53 pathway status. Using user-defined criteria, the system identified late-onset PDAC patients not treated with gemcitabine and partitioned them into TP53 pathway-altered (*n* = 47) and non-altered (*n* = 26) groups. Kaplan–Meier analysis revealed a statistically significant difference in overall survival between these cohorts (log-rank *p* = 0.0113), with TP53 pathway alterations associated with inferior outcomes. This example highlights the ability of conversational AI to reproducibly generate clinically relevant subgroups and perform survival modeling within narrowly defined contexts.

We next applied the AI-HOPE-TP53 agent to investigate the relationship between gene-level TP53 mutations and broader pathway-level dysregulation (Figure S2). Restricting the analysis to tumors harboring TP53 mutations, the system constructed comparison cohorts based on the presence or absence of additional TP53 pathway alterations. Contingency-based statistical testing demonstrated a highly significant enrichment of pathway-level alterations among TP53-mutant tumors (*p* ≈ 0), with a strong odds ratio indicating substantial overlap between mutation status and pathway activation. This analysis underscores the dominant role of TP53 as the central node within its pathway and illustrates how conversational AI can quantify hierarchical relationships between gene-level events and pathway-level architecture.

AI-driven exploratory analyses highlight the utility of conversational frameworks for interrogating clinically stratified datasets. Through dynamic cohort definition, integrated survival analysis, and enrichment testing, AI-HOPE-TP53 enables efficient hypothesis generation and validation across complex clinical and molecular contexts.

#### 2.6.2. AI-HOPE-PI3K: Contextual Analysis of PI3K Pathway Associations

We next applied the AI-HOPE-PI3K agent to systematically explore clinical and genomic correlates of PI3K pathway alterations across the PDAC cohort, with particular emphasis on treatment-associated patterns and pathway-level interactions (Figures S3 and S4).

To establish a global view of PI3K pathway-associated features, the agent first constructed cohorts stratified by PI3K pathway status (Figure S3). Tumors harboring PI3K pathway alterations (*n* = 16) were compared with a substantially larger group lacking such alterations (*n* = 168), reflecting the relatively low prevalence of PI3K dysregulation in this dataset. Through automated Chi-square-based association testing, the AI framework identified significant relationships between PI3K pathway status and multiple components of the PI3K signaling axis, including PIK3CA, STK11, MTOR, PPP2R1A, RICTOR, TSC2, and RPTOR. These findings indicate coordinated perturbation across upstream regulators and mTOR complex-related nodes rather than reliance on a single dominant alteration.

Beyond canonical pathway members, the analysis also revealed associations with additional signaling and regulatory genes, including NLK, RNF43, and TCF7, as well as calcium channel-related genes within the CACNA family. Enrichment of WNT pathway alterations further suggests cross-talk between PI3K signaling and other oncogenic networks. Clinically, PI3K pathway status was associated with gemcitabine treatment exposure and progression-free status, and differences were also observed across histologic subtypes. Together, these results define PI3K-altered PDAC as a distinct molecular subset characterized by multi-node pathway involvement and broader signaling network integration.

We then investigated whether PI3K pathway alterations were preferentially associated with gemcitabine exposure using an AI-driven contingency analysis (Figure S4). The agent compared treatment distribution between PI3K-altered and PI3K-wild-type tumors, demonstrating a significantly higher proportion of gemcitabine-treated cases among PI3K-altered tumors (87.5% vs. 54.76%). Statistical testing confirmed this association (*p* = 0.023), with an elevated odds ratio indicating enrichment of PI3K pathway alterations in the treatment-exposed population.

This finding supports a model in which PI3K pathway dysregulation may be linked to treatment context, potentially reflecting differences observed across treatment contexts or adaptive signaling responses. Importantly, the AI-HOPE-PI3K framework enhanced the rapid identification of this relationship through structured query execution and automated statistical analyses, highlighting its utility for uncovering clinically relevant, treatment-associated molecular patterns.

PI3K pathway alterations in PDAC exhibit both genomic heterogeneity and context-dependent associations with treatment exposure and signaling networks. The AI-driven approach enables efficient integration of molecular and clinical data, offering insights into treatment-dependent disease biology.

## 3. Discussion

PDAC remains a biologically aggressive and clinically heterogeneous malignancy in which treatment response is shaped by both intrinsic tumor genetics and the context in which those alterations operate. In this study, we applied a conversational artificial intelligence strategy, using AI-HOPE-TP53 [33] and AI-HOPE-PI3K [34], to interrogate TP53- and PI3K-centered pathway architecture across a clinically stratified PDAC cohort defined by age at diagnosis and gemcitabine exposure. Several key findings emerged from this analysis. First, TP53 pathway disruption was common across the cohort, but gene-level analysis revealed that TP53 mutations were particularly enriched in gemcitabine-treated early-onset PDAC. Second, PI3K pathway alterations were much less frequent overall, yet they were significantly enriched in gemcitabine-treated late-onset PDAC and displayed a broader, more distributed mutational architecture in that setting. Third, the prognostic value of both pathways was highly context dependent, with the clearest survival differences observed among late-onset patients who had not received gemcitabine, where the absence of either TP53 or PI3K pathway alterations identified more favorable-outcome subgroups.

A central message of this work is that pathway prevalence alone does not fully capture clinically meaningful biology. At the aggregate level, TP53 pathway alterations appeared broadly stable across age and treatment categories, which could suggest that TP53 dysfunction is simply a ubiquitous background feature of PDAC. However, deeper inspection at the gene level showed that this apparent stability conceals meaningful differences in the distribution of the dominant event within the pathway, namely TP53 mutation itself. In particular, the significantly higher TP53 mutation frequency in gemcitabine-treated early-onset tumors relative to gemcitabine-treated late-onset tumors suggests that early-onset PDAC may exhibit enrichment of TP53-driven biology within gemcitabine-exposed clinical contexts. This does not necessarily imply that gemcitabine induces TP53 alterations, but it raises the possibility that gemcitabine-exposed early-onset tumors may be enriched for TP53-disrupted molecular states associated with aggressive disease biology.

This observation is biologically plausible. TP53 loss is tightly linked to impaired apoptosis, defective cell-cycle checkpoint control, and increased tolerance of genotoxic stress, all of which may influence response to cytotoxic therapy. In PDAC, where treatment resistance is often multifactorial, higher TP53 mutation rates in gemcitabine-treated early-onset tumors may reflect a disease subset better equipped to survive DNA damage and metabolic stress. The trend toward higher TP53 mutation frequency in treated versus untreated early-onset disease, although not statistically significant, further supports this interpretation and suggests that a larger cohort may reveal a more robust treatment-associated signal in younger patients. By contrast, late-onset disease showed similar TP53 mutation frequencies regardless of gemcitabine exposure, implying that TP53-driven biology in older patients may be more constitutive and less specifically shaped by this treatment context.

The PI3K pathway showed a very different pattern. Unlike TP53, PI3K pathway alterations were uncommon at the cohort level, but their distribution was more clinically informative. The significant enrichment of PI3K pathway alterations in gemcitabine-treated late-onset PDAC points to a treatment-contextual expansion of this signaling axis in older patients. Importantly, this enrichment was not driven by a single dominant gene. Rather, the pathway in treated late-onset tumors was characterized by low-frequency alterations spread across multiple nodes, including PIK3CA, PIK3R family members, AKT isoforms, TSC1/2, STK11, RICTOR, MTOR, and related regulators. This kind of dispersed architecture suggests that PI3K pathway activation in PDAC may arise through several parallel or partially redundant molecular routes, especially under therapeutic pressure.

That pattern is conceptually important. A pathway dominated by one highly recurrent event often implies a central, shared dependency. In contrast, a pathway altered through many different low-frequency lesions may reflect adaptive plasticity rather than a single essential driver. In this study, the broader mutational distribution seen in gemcitabine-treated late-onset PDAC is consistent with the idea that PI3K pathway heterogeneity may reflect context-dependent signaling diversity associated with treatment exposure. Even though individual PI3K-axis genes did not show statistically significant differences on their own, the collective diversification of the pathway in treated late-onset disease is difficult to dismiss as random noise, particularly because the pathway-level enrichment was significant. These patterns are consistent with potential differences in signaling architecture across treatment contexts, although mechanistic studies are required to determine whether these observations reflect therapy-associated selection, baseline biological heterogeneity, or other clinical factors.

Another important finding is that the prognostic relevance of both pathways was concentrated in late-onset patients who did not receive gemcitabine. In that subgroup, absence of TP53 pathway alterations was associated with significantly better overall survival, and the same was true, even more strongly, for the absence of PI3K pathway alterations. By contrast, in gemcitabine-treated patients and in early-onset disease, neither pathway consistently stratified survival. This suggests that the prognostic meaning of pathway status is not fixed, but instead depends on treatment exposure and disease context.

Several explanations may account for this pattern. In untreated late-onset disease, pathway alterations may more directly reflect the baseline natural history of the tumor, unmodified by the confounding effects of systemic therapy. In that setting, tumors lacking TP53 or PI3K pathway disruption may represent biologically less aggressive subsets or may rely on alternate oncogenic programs associated with slower progression. Conversely, once patients are exposed to gemcitabine, treatment-related selection, line-of-therapy effects, and unmeasured clinical confounders may dilute or obscure the prognostic signal attributable to any single pathway. This may be especially relevant in PDAC, where treatment decisions often correlate with fitness, stage, comorbidity, and other factors that are incompletely captured in retrospective clinicogenomic datasets. Accordingly, differences observed between gemcitabine-exposed and non-exposed groups should not be interpreted as causal effects of therapy on pathway evolution.

The survival findings also reinforce a broader conceptual point: prognostic markers and predictive markers are not interchangeable. The enrichment of PI3K pathway alterations in gemcitabine-treated late-onset PDAC does not automatically mean PI3K status predicts gemcitabine response, just as the adverse survival associated with TP53 or PI3K alterations in untreated late-onset disease does not necessarily establish those pathways as treatment-selection biomarkers. Rather, the present data indicate that these pathways carry different forms of clinical information depending on setting. In some contexts they appear to track tumor aggressiveness, while in others they may reflect treatment-associated evolutionary pressure. Distinguishing those roles will require datasets with more granular therapy timing, response data, and multivariable modeling. Importantly, the present analyses were designed to evaluate associations between pathway status and clinical outcomes within stratified subgroups and were not intended to establish predictive biomarkers of gemcitabine response.

Methodologically, this study also demonstrates the practical value of conversational artificial intelligence as an analytic interface for precision oncology. AI-HOPE-TP53 and AI-HOPE-PI3K enabled rapid cohort construction, stratified pathway interrogation, and hypothesis generation across multiple overlapping clinical dimensions without abandoning reproducibility. This is particularly useful in studies like this one, where clinically relevant subgrouping is not simple. Investigators may need to move repeatedly between age-defined, treatment-defined, pathway-level, gene-level, and survival-based analyses. Conversational AI does not replace statistical rigor, but it can substantially reduce friction in exploratory and semi-structured analysis, allowing complex questions to be posed and iteratively refined in ways that are more aligned with translational reasoning. The fact that AI-derived findings were validated using conventional statistical approaches strengthens the case that such systems can serve as a credible analytic layer rather than a black-box shortcut. Because several subgroup analyses involved limited sample sizes, particularly in early-onset disease, these survival observations should be considered exploratory and hypothesis-generating rather than definitive prognostic stratifications.

These results should be interpreted in light of several limitations. The most important is the relatively small and imbalanced size of several clinically stratified subgroups, particularly the early-onset non-gemcitabine-treated cohort (*n* = 5) and the PI3K-altered subgroup relative to PI3K-wild-type tumors. These imbalances reduce statistical power, widen confidence intervals, and increase susceptibility to unstable or potentially spurious associations, especially in subgroup-specific survival and gene-level analyses. Accordingly, the present findings should be interpreted as exploratory and hypothesis-generating rather than definitive evidence of biologically distinct molecular subclasses. Second, gemcitabine exposure was modeled as a simplified binary variable, whereas real-world PDAC treatment is substantially more complex and heterogeneous. Detailed information regarding combination chemotherapy regimens, treatment sequencing, cumulative dose intensity, treatment duration, line-of-therapy effects, and exact temporal relationships between systemic therapy and molecular profiling was not uniformly available across the cohort. As a result, observed associations involving gemcitabine-exposed tumors should not be interpreted as direct treatment-induced molecular effects. Instead, these findings more appropriately reflect molecular patterns observed within clinically treatment-defined subgroups. Third, the cohort was dominated by stage II disease, which may limit generalizability to more advanced PDAC populations. Fourth, DNA-level pathway alteration status does not directly measure pathway activity. A tumor without a mutation in a PI3K pathway gene may still have functional pathway activation through epigenetic, transcriptional, post-translational, stromal, or metabolic mechanisms. Similarly, not all mutations within a pathway are functionally equivalent. Fifth, the demographic composition of the cohort limits equity-oriented interpretation. Hispanic/Latino cases were sparse and ethnicity was unknown in a sizable fraction of patients, preventing meaningful evaluation of ancestry- or ethnicity-associated differences in TP53 or PI3K biology. Given the retrospective and observational nature of the study, all identified relationships should be interpreted as associative rather than causal.

An additional limitation of this study relates to subgroup-specific survival analyses. We did not perform multivariable Cox proportional hazards modeling because several clinically stratified cohorts, particularly early-onset and PI3K-altered subgroups, contained limited sample sizes and low event counts, increasing the risk of model overfitting and unstable hazard ratio estimates. In addition, important treatment-related variables, including combination regimens, therapy sequencing, dose intensity, and timing relative to molecular profiling, were not uniformly available across the retrospective dataset. Accordingly, Kaplan–Meier and log-rank analyses were used as exploratory descriptive approaches, and the survival findings should be interpreted as hypothesis-generating rather than definitive independent prognostic associations.

The analyses were restricted to DNA-level genomic alterations derived from publicly available clinicogenomic datasets, as matched transcriptomic, proteomic, spatial, and tumor microenvironment data were not consistently available across the cohort. Consequently, pathway alteration status should not be interpreted as a direct measure of functional pathway activation. Integration of additional molecular layers, including transcriptomics, phosphoproteomics, spatial biology, and immune microenvironment characterization, would likely improve biological resolution and predictive modeling by capturing context-dependent signaling activity beyond genomic alterations alone. Future multi-omics studies incorporating these complementary data modalities will be important to more comprehensively define TP53- and PI3K-associated molecular states in PDAC and to refine clinically relevant precision oncology stratification.

A further limitation of this study is the absence of KRAS-adjusted analyses. KRAS alterations represent a central and highly prevalent molecular feature of PDAC biology and may influence both TP53- and PI3K-associated signaling contexts and downstream pathway interactions. Because KRAS mutations are relatively ubiquitous in PDAC, the present exploratory analyses focused specifically on TP53- and PI3K-centered pathway architecture without stratifying by KRAS co-mutation status. Consequently, some of the observed molecular and survival associations may partially reflect broader KRAS-driven biological dependencies or co-mutational signaling interactions not captured in the current study design. Future investigations incorporating integrated co-mutation modeling, multivariable pathway interaction analyses, and broader signaling-network interrogation will be important to more comprehensively define the context-dependent biological relationships underlying TP53 and PI3K pathway alterations in PDAC.

One important consideration is the potential technical heterogeneity introduced through the integration of sequencing data obtained from multiple independent sources. Although molecular data were harmonized to improve consistency in variant annotation, pathway assignment, and downstream comparative analyses, differences in sequencing platforms, panel composition, coverage depth, bioinformatic processing pipelines, variant-calling algorithms, and reporting standards across contributing datasets may introduce residual technical variability that cannot be fully excluded in retrospective integrative analyses. Such differences may influence mutation detection sensitivity, low-frequency variant identification, and pathway-level classification, potentially contributing to variability across clinically stratified subgroups. Future studies using prospectively standardized sequencing methodologies and harmonized bioinformatic workflows will be important to minimize technical heterogeneity and further validate the reproducibility of these findings.

A further caveat of the present study is the incomplete availability of comprehensive treatment annotation across the retrospective cohort. Detailed information regarding concomitant therapies, including radiation therapy, non-gemcitabine chemotherapy regimens, targeted therapies, immunotherapy exposure, treatment sequencing, cumulative dose intensity, and duration of therapy, was not uniformly available for all patients included in the analysis. As a result, treatment exposure was simplified into a binary gemcitabine-exposed versus non-exposed classification to support clinically stratified exploratory analyses. Consequently, the observed molecular and survival associations should not be interpreted as isolated effects of gemcitabine exposure alone, as additional unmeasured treatment-related variables and clinical confounders may have contributed to the observed patterns. Future studies incorporating more detailed longitudinal treatment annotation and time-matched molecular profiling will be important to more accurately define treatment-specific molecular dependencies in PDAC.

An additional challenge associated with this analysis is that OS was used as the primary clinical endpoint because progression-free survival, disease-specific survival, recurrence-free survival, and uniformly annotated recurrence data were not consistently available across the publicly curated datasets included in this study. Although OS provides a broadly accessible and clinically relevant outcome measure, it may be influenced by non-cancer-related mortality, comorbidities, and treatment heterogeneity that are not directly attributable to PDAC biology. Consequently, the observed survival associations may not fully capture disease-specific molecular behavior or treatment response dynamics. Future studies incorporating more comprehensive longitudinal clinical annotation and disease-specific outcome metrics will be important to better define the prognostic and biological significance of TP53 and PI3K pathway alterations in PDAC.

Future work should build on these observations in several directions. Validation in larger and independent PDAC cohorts is essential, particularly to confirm the enrichment of TP53 mutations in gemcitabine-treated early-onset disease and the context-dependent pathway heterogeneity of PI3K signaling in late-onset PDAC. Because PI3K pathway alterations occurred in a relatively small proportion of tumors, pathway-specific associations and survival differences involving PI3K-altered cases require independent validation in larger cohorts. More detailed treatment annotation will help determine whether these are truly chemotherapy-associated features or reflections of broader disease selection. Functional studies are also needed. For TP53, this means testing whether early-onset, TP53-mutant PDAC shows distinct apoptotic thresholds, genomic instability profiles, or chemotherapy adaptation states. For PI3K, it will be important to determine whether the distributed set of low-frequency alterations identified in treated late-onset tumors converges on shared downstream signaling outputs that could still be therapeutically targetable.

In summary, this study supports a model in which TP53 and PI3K pathways contribute to PDAC biology in distinct but clinically meaningful ways. TP53 alterations form a dominant and recurrent backbone of disease, but their enrichment in gemcitabine-treated early-onset PDAC suggests that age and treatment can shape even highly recurrent tumor suppressor events. PI3K alterations, in contrast, are infrequent but more plastic, and their significant enrichment in gemcitabine-treated late-onset tumors points to a context in which signaling diversity may expand under treatment pressure. The strongest prognostic effects for both pathways were seen in late-onset patients not treated with gemcitabine, indicating that pathway status can stratify survival most clearly when not obscured by treatment-related confounding. Together, these findings argue for moving beyond simple pathway-present versus pathway-absent classification and toward clinically contextualized, gene-level dependency mapping. They also illustrate how conversational AI can accelerate that process by making complex, multidimensional molecular analyses more accessible, reproducible, and hypothesis-generating within a precision oncology framework. Conversational AI served primarily as an interface for structured cohort interrogation and iterative exploratory analysis, while all statistical findings were independently validated using conventional analytical workflows.

### Conclusions

In this clinically stratified PDAC cohort, TP53 and PI3K pathways showed distinct patterns of molecular organization and clinical relevance. TP53 pathway disruption was common across the cohort, but TP53 mutations were particularly enriched in gemcitabine-treated early-onset PDAC. In contrast, PI3K pathway alterations were significantly enriched in gemcitabine-treated late-onset disease and were characterized by a broader, low-frequency mutational spectrum distributed across multiple pathway components. Exploratory survival analyses suggested that the absence of TP53 or PI3K pathway alterations identified favorable-prognosis subsets within late-onset PDAC patients who did not receive gemcitabine.

These findings suggest that age at onset and treatment exposure may influence the clinical and molecular context in which TP53 and PI3K pathway alterations are observed in PDAC. They also highlight the value of conversational artificial intelligence as a practical platform for context-aware, pathway-centric interrogation of clinicogenomic data. By enabling rapid and reproducible analysis of clinically stratified molecular subgroups, AI-HOPE-TP53 and AI-HOPE-PI3K support a more nuanced precision oncology approach to this highly lethal disease.

## 4. Materials and Methods

### 4.1. Study Design, Data Integration, and Clinical Stratification

This study was designed as a retrospective, integrative clinicogenomic analysis of PDAC aimed at characterizing TP53 and PI3K pathway alterations across clinically relevant contexts defined by age at diagnosis and gemcitabine exposure. A total of 184 PDAC tumor specimens with matched clinical and molecular annotations were included in the final analytical cohort. Clinical variables included age at diagnosis, sex, disease stage, treatment exposure, specimen source, ethnicity, survival status, and follow-up information. Molecular data were obtained from tumor-based next-generation sequencing (NGS) platforms and harmonized across datasets to ensure consistency in variant annotation, pathway assignment, and downstream comparative analyses.

To maintain patient-level independence and avoid overrepresentation of individuals with multiple sequencing records, only one tumor profile per patient was retained for final analysis. In cases where multiple sequencing assays or repeated molecular profiles were available for the same individual, a predefined prioritization strategy was applied. Preference was given to: (i) samples with the most comprehensive genomic coverage, (ii) primary tumor specimens over metastatic lesions when both were available, and (iii) molecular profiles temporally aligned with documented treatment exposure whenever sequencing timing information was available. This approach minimized potential confounding associated with tumor evolution, treatment-induced clonal selection, or repeated sampling.

All tumor specimens included in the study originated from primary PDAC lesions, thereby reducing biological heterogeneity introduced by metastatic-site-specific genomic divergence. Clinical and molecular datasets were integrated through standardized patient-level identifiers and subjected to quality-control review to ensure consistency across demographic, clinical, and genomic annotations.

Patients were stratified into clinically defined subgroups based on age at diagnosis and gemcitabine exposure. Early-onset PDAC was defined as diagnosis before 50 years of age, whereas late-onset PDAC was defined as diagnosis at or after 50 years of age. This threshold was selected based on prior literature supporting biologically and clinically distinct features of early-onset PDAC. Treatment exposure was classified using documented systemic therapy records. Treatment exposure was defined using available systemic therapy annotations extracted from clinical records. Patients were categorized as gemcitabine-exposed if they received gemcitabine-containing regimens at any point during their documented clinical course and as non-exposed otherwise. Because treatment data were retrospectively derived and not uniformly standardized across all patients, detailed information regarding combination regimens, treatment sequencing, cumulative dose intensity, duration of therapy, line of treatment, and exact timing of molecular profiling relative to therapy administration was not consistently available for all cases. When temporal treatment information was available, sequencing timepoints were aligned with documented therapy exposure to improve concordance between clinical treatment context and molecular profiling. Accordingly, gemcitabine exposure was modeled as a simplified binary clinical variable intended to support exploratory stratified analyses rather than comprehensive treatment-response modeling.

The resulting cohort was subsequently subdivided into four clinically relevant analytical groups: (i) early-onset gemcitabine-treated PDAC, (ii) early-onset non-gemcitabine-treated PDAC, (iii) late-onset gemcitabine-treated PDAC, and (iv) late-onset non-gemcitabine-treated PDAC. These stratifications enabled context-specific pathway analyses evaluating the interaction between tumor biology, age, and treatment exposure.

### 4.2. Pathway Definition, Genomic Annotation, and Variant Filtering

Two biologically informed pathway-centered gene panels were curated for analysis: (i) the TP53-associated tumor suppressor signaling network and (ii) the PI3K/AKT/mTOR signaling axis. Gene selection was based on established biological roles in PDAC pathogenesis, DNA damage response, apoptosis, cell-cycle regulation, cell survival signaling, metabolic adaptation, and therapeutic resistance.

The TP53 pathway panel included TP53 and functionally related genes implicated in genomic stability, checkpoint regulation, apoptosis, and DNA damage signaling. The PI3K pathway panel included catalytic subunits, regulatory components, upstream regulators, downstream effectors, and mTOR complex-associated genes involved in PI3K/AKT signaling dynamics.

Somatic variants were extracted from harmonized NGS datasets and subjected to standardized filtering procedures prior to pathway-level aggregation. Only protein-altering variants with predicted functional relevance were retained for analysis. Qualifying alterations included missense mutations, nonsense mutations, frameshift insertions or deletions, splice-site alterations, start-loss variants, and truncating mutations. Synonymous variants, deep intronic variants, untranslated-region variants, and non-coding alterations lacking predicted functional impact were excluded. Variants annotated as likely benign or without sufficient evidence of pathogenicity were not incorporated into pathway-level alteration definitions.

Pathway alteration status was defined at the patient level by the presence of at least one qualifying somatic alteration within the corresponding pathway gene set. Gene-level alteration frequencies were subsequently calculated within each clinically stratified subgroup to evaluate context-specific mutational distributions. Because the PI3K pathway is biologically distributed across multiple signaling nodes rather than dominated by a single recurrent alteration, pathway-level aggregation was used to capture broader signaling-axis perturbation beyond individual gene frequencies.

### 4.3. Statistical Analysis and Survival Modeling

The primary molecular objective of the study was to evaluate differences in TP53 and PI3K pathway alteration frequencies across age- and treatment-defined PDAC subgroups. Comparisons of categorical variables were performed using Fisher’s exact test or Pearson’s chi-square test, as appropriate based on subgroup sample size and expected contingency-table frequencies. All statistical tests were two-sided, and statistical significance was defined as a *p*-value < 0.05.

Descriptive statistics were used to summarize baseline demographic, clinical, and molecular characteristics of the cohort. Continuous variables were summarized using medians and ranges when appropriate, whereas categorical variables were summarized as frequencies and percentages.

Overall survival (OS) was defined as the interval from date of diagnosis to death or last documented follow-up. Survival analyses were conducted using the Kaplan–Meier method, and differences between survival curves were assessed using the log-rank test. Subgroup-specific survival analyses were performed within clinically stratified contexts to evaluate the prognostic impact of TP53 and PI3K pathway alterations under distinct age and treatment conditions.

Because several clinically stratified subgroups contained relatively small sample sizes, survival analyses were interpreted cautiously, particularly in early-onset PDAC subsets. Confidence intervals were evaluated alongside survival estimates to assess the stability of subgroup-specific observations. The study was designed primarily as a hypothesis-generating exploratory analysis rather than a definitive predictive biomarker study. Given the exploratory nature of clinically stratified subgroup analyses and the limited sample sizes of certain cohorts, particularly early-onset and PI3K-altered subsets, statistical findings were interpreted cautiously and considered hypothesis-generating.

All statistical analyses were independently validated using conventional computational workflows implemented outside the AI platform. Statistical outputs generated through the conversational AI framework were cross-checked against manually executed analytical pipelines to confirm concordance in cohort assignment, mutation frequency calculations, contingency testing, and survival modeling. This dual-validation strategy was implemented to ensure methodological rigor, reproducibility, and analytical transparency.

### 4.4. Artificial Intelligence-Enabled Analytical Framework

To enable scalable interrogation of multidimensional clinicogenomic relationships, we implemented a conversational artificial intelligence framework based on the AI-HOPE platform. This framework supports natural language-driven cohort construction and dynamic pathway interrogation across integrated clinical and genomic datasets.

Specialized pathway-centered modules, including AI-HOPE-TP53 and AI-HOPE-PI3K, were utilized to perform exploratory analyses involving mutation frequency aggregation, subgroup comparisons, enrichment analyses, pathway-level association testing, and survival modeling. The system translates user-defined natural language queries into structured analytical workflows, enabling rapid iterative hypothesis generation across clinically relevant strata.

The AI framework enabled construction of highly specific analytical cohorts defined simultaneously by age, treatment exposure, molecular alteration status, and survival characteristics. This functionality facilitated flexible exploration of clinically contextualized pathway dependencies that would otherwise require multiple sequential manual analyses.

Importantly, the conversational AI framework was used as an analytical interface rather than a replacement for statistical validation. All AI-generated cohort definitions, contingency analyses, mutation frequencies, odds ratios, and Kaplan–Meier outputs were independently reproduced using conventional statistical pipelines. Concordance between AI-generated and manually validated outputs was systematically evaluated to confirm analytical reproducibility, data integrity, and methodological transparency.

This hybrid strategy combining conversational AI-guided exploration with conventional statistical validation was designed to preserve interpretability and reproducibility while enabling efficient interrogation of complex clinicogenomic relationships in PDAC.

To support methodological transparency and reproducibility, additional technical information regarding the AI-HOPE conversational artificial intelligence framework was made publicly available through the AI-HOPE GitHub repository. The repository includes documentation describing the analytical workflow, natural language-driven cohort construction strategy, pathway-centric query structure, implementation framework, and computational pipelines used for exploratory clinicogenomic analyses. The GitHub resource also provides information regarding system architecture, reproducible analytical procedures, and example query execution workflows relevant to the AI-HOPE platform. This resource was included to facilitate methodological accessibility, reproducibility, and future development of conversational AI applications in precision oncology research.

## Figures and Tables

**Table 1:** Overview of Baseline Demographic, Clinical, and Genomic Characteristics of the Study Cohort.

Category	Subgroup	*n* (%)
Age at Onset and Treatment Status	Early-Onset (<50), Gemcitabine-Treated	15 (8.2%)
	Late-Onset (≥50), Gemcitabine-Treated	91 (49.5%)
	Early-Onset (<50), Non-Gemcitabine-Treated	5 (2.7%)
	Late-Onset (≥50), Non-Gemcitabine-Treated	73 (39.7%)
Tumor Classification	Pancreatic Adenocarcinoma	184 (100.0%)
Sex Distribution	Male	101 (54.9%)
	Female	83 (45.1%)
Specimen Type	Primary Tumor	184 (100.0%)
Stage at Diagnosis	Stage I	21 (11.4%)
	Stage II	151 (82.1%)
	Stage III	5 (2.7%)
	Stage IV	5 (2.7%)
	Not Available	2 (1.1%)
Ethnicity	Hispanic or Latino	5 (2.7%)
	Not Hispanic or Latino	136 (73.9%)
	Unknown	43 (23.4%)

**Table 2:** Distribution of TP53 and PI3K Pathway Alterations Across Age- and Treatment-Stratified PDAC Subgroups. This table summarizes the frequency of TP53 and PI3K pathway alterations across pancreatic ductal adenocarcinoma (PDAC) cohorts stratified by age at diagnosis (early-onset vs. late-onset) and gemcitabine exposure. The analysis is organized into four complementary panels to facilitate pathway-specific and clinically contextual interpretation: (**a**) TP53 pathway alterations comparing gemcitabine-treated and non-gemcitabine-treated tumors within early-onset PDAC; (**b**) TP53 pathway alterations comparing early-onset and late-onset disease within each treatment group; (**c**) PI3K pathway alterations comparing gemcitabine-treated and non-treated tumors within early-onset PDAC; and (**d**) PI3K pathway alterations comparing early- and late-onset PDAC within each treatment stratum.

(a)						
Pathway Alterations	Early-Onset Treated with Gemcitabine *n* (%)	Early-Onset Not Treated with Gemcitabine *n* (%)	*p*-Value	Late-Onset Treated with Gemcitabine *n* (%)	Late-Onset Not Treated with Gemcitabine *n* (%)	*p*-Value
TP53 Alterations Present	12 (80.0%)	2 (40.0%)	0.1313	57 (62.6%)	47 (64.4%)	0.9461
TP53 Alterations Absent	3 (20.0%)	3 (60.0%)		34 (37.4%)	26 (35.6%)	
(**b**)						
**Pathway Alterations**	**Early-Onset** **Treated with Gemcitabine** ***n* (%)**	**Late-Onset** **Treated with Gemcitabine** ***n* (%)**	***p*-value**	**Early-Onset** **Not Treated with Gemcitabine** ***n* (%)**	**Late-Onset** **Not Treated with Gemcitabine** ***n* (%)**	***p*-value**
TP53 Alterations Present	12 (80.0%)	57 (62.6%)	0.2496	2 (40.0%)	47 (64.4%)	0.3546
TP53 Alterations Absent	3 (20.0%)	34 (37.4%)		3 (60.0%)	26 (35.6%)	
(**c**)						
**Pathway Alterations**	**Early-Onset** **Treated with Gemcitabine** ***n* (%)**	**Early-Onset** **Not Treated with Gemcitabine** ***n* (%)**	***p*-value**	**Late-Onset** **Treated with Gemcitabine** ***n* (%)**	**Late-Onset** **Not Treated with Gemcitabine** ***n* (%)**	***p*-value**
PI3K Alterations Present	2 (13.3%)	0 (0.0%)	1	12 (13.2%)	2 (2.7%)	**0.02266**
PI3K Alterations Absent	13 (86.7%)	5 (100.0%)		79 (86.8%)	71 (97.3%)	
(**d**)						
**Pathway Alterations**	**Early-Onset** **Treated with Gemcitabine** ***n* (%)**	**Late-Onset** **Treated with Gemcitabine** ***n* (%)**	***p*-value**	**Early-Onset** **Not Treated with Gemcitabine** ***n* (%)**	**Late-Onset** **Not Treated with Gemcitabine** ***n* (%)**	***p*-value**
PI3K Alterations Present	2 (13.3%)	12 (13.2%)	1	0 (0.0%)	2 (2.7%)	**1**
PI3K Alterations Absent	13 (86.7%)	79 (86.8%)		5 (100.0%)	71 (97.3%)	

## Data Availability

The data presented in this study are openly available in [cBioPortal at https://www.cbioportal.org (accessed on 12 March 2026)] and [genie cBioportal at https://genie.cbioportal.org/ (accessed on 12 March 2026)]. Analytical resources are available through the GitHub repository https://github.com/Velazquez-Villarreal-Lab/AI-TP53 (accessed on 12 March 2026) and https://github.com/Velazquez-Villarreal-Lab/AI-PI3K (accessed on 12 March 2026) to promote transparency and reproducibility. Additional data can be provided by the authors upon reasonable request.

## Supplementary Materials

The following supporting information can be downloaded at: https://www.mdpi.com/article/10.3390/ijms27114981/s1.
