# Supplementary material for: Age- and Treatment-Specific TP53 and PI3K Alterations in Pancreatic Ductal Adenocarcinoma (PDAC) Revealed by Conversational Artificial Intelligence
Source: Int J Mol Sci. 2026 May 30;27(11):4981. doi: 10.3390/ijms27114981 (PMC13257402; doi:10.3390/ijms27114981)
Supplement: Supplementary file 1 [file ijms-27-04981-s001.zip › ijms-4277970-supplementary.pdf]

Supplementary Materials:

Table S1. Comparison of Early-Onset PDAC Patients Treated with Gemcitabine Versus Those Not Treated with Gemcitabine.

TP53 Pathway			
**Gene**	**Early-Onset Treated with Gemcitabine n (%)**	**Early-Onset Not Treated with Gemcitabine n (%)**	**p-value**
**TP53 Mutation**			
Present	13 (86.7%)	2 (40.0%)	0.07263
Absent	2 (13.3%)	3 (60.0%)	
**MDM2 Mutation**			
Present	0 (0.0%)	0 (0.0%)	1
Absent	15 (100.0%)	5 (100.0%)	
**MDM4 Mutation**			
Present	0 (0.0%)	0 (0.0%)	1
Absent	15 (100.0%)	5 (100.0%)	
**CDKN1A Mutation**			
Present	0 (0.0%)	0 (0.0%)	1
Absent	15 (100.0%)	5 (100.0%)	
**CDKN2A Mutation**			
Present	2 (13.3%)	1 (20.0%)	1
Absent	13 (86.7%)	4 (80.0%)	
**ATM Mutation**			
Present	0 (0.0%)	0 (0.0%)	1
Absent	15 (100.0%)	5 (100.0%)	
**CHEK1 Mutation**			
Present	0 (0.0%)	0 (0.0%)	1
Absent	15 (100.0%)	5 (100.0%)	
**CHEK2 Mutation**			
Present	0 (0.0%)	0 (0.0%)	1
Absent	15 (100.0%)	5 (100.0%)	
**PTEN Mutation**			
Present	0 (0.0%)	0 (0.0%)	1
Absent	15 (100.0%)	5 (100.0%)	
**ATR Mutation**			
Present	1 (6.7%)	0 (0.0%)	1
Absent	14 (93.3%)	5 (100.0%)	
**BBC3 Mutation**			

Present	0 (0.0%)	0 (0.0%)	1
Absent	15 (100.0%)	5 (100.0%)	

Table S2. Comparison of Late-Onset PDAC Patients Treated with Gemcitabine Versus Those Not Treated with Gemcitabine.

TP53 Pathway			
**Gene**	**Late-Onset Treated with Gemcitabine n (%)**	**Late-Onset Not Treated with Gemcitabine n (%)**	**p-value**
**TP53 Mutation**			
Present	52 (57.1%)	44 (60.3%)	0.8064
Absent	39 (42.9%)	29 (39.7%)	
**MDM2 Mutation**			
Present	1 (1.1%)	1 (1.4%)	1
Absent	90 (98.9%)	72 (98.6%)	
**MDM4 Mutation**			
Present	0 (0.0%)	0 (0.0%)	1
Absent	91 (100.0%)	73 (100.0%)	
**CDKN1A Mutation**			
Present	0 (0.0%)	0 (0.0%)	1
Absent	91 (100.0%)	73 (100.0%)	
**CDKN2A Mutation**			
Present	18 (19.8%)	14 (19.2%)	1
Absent	73 (80.2%)	59 (80.8%)	
**ATM Mutation**			
Present	5 (5.5%)	3 (4.1%)	0.7334
Absent	86 (94.5%)	70 (95.9%)	
**CHEK1 Mutation**			
Present	0 (0.0%)	0 (0.0%)	1
Absent	91 (100.0%)	73 (100.0%)	
**CHEK2 Mutation**			
Present	1 (1.1%)	0 (0.0%)	1
Absent	90 (98.9%)	73 (100.0%)	
**PTEN Mutation**			
Present	0 (0.0%)	0 (0.0%)	1
Absent	91 (100.0%)	73 (100.0%)	
**ATR Mutation**			
Present	2 (2.2%)	1 (1.4%)	1

Absent	89 (97.8%)	72 (98.6%)	
**BBC3 Mutation**			
Present	0 (0.0%)	0 (0.0%)	1
Absent	91 (100.0%)	73 (100.0%)	

Table S3. Comparison of Early-Onset PDAC Patients Versus Late-Onset PDAC Patients Treated with Gemcitabine.

**TP53 Pathway**			
**Gene**	**Early-Onset Treated with Gemcitabine n (%)**	**Late-Onset Treated with Gemcitabine n (%)**	**p-value**
**TP53 Mutation**			
Present	13 (86.7%)	52 (57.1%)	**0.04312**
Absent	2 (13.3%)	39 (42.9%)	
**MDM2 Mutation**			
Present	0 (0.0%)	1 (1.1%)	1
Absent	15 (100.0%)	90 (98.9%)	
**MDM4 Mutation**			
Present	0 (0.0%)	0 (0.0%)	1
Absent	15 (100.0%)	91 (100.0%)	
**CDKN1A Mutation**			
Present	0 (0.0%)	0 (0.0%)	1
Absent	15 (100.0%)	91 (100.0%)	
**CDKN2A Mutation**			
Present	2 (13.3%)	18 (19.8%)	0.7312
Absent	13 (86.7%)	73 (80.2%)	
**ATM Mutation**			
Present	0 (0.0%)	5 (5.5%)	1
Absent	15 (100.0%)	86 (94.5%)	
**CHEK1 Mutation**			
Present	0 (0.0%)	0 (0.0%)	1
Absent	15 (100.0%)	91 (100.0%)	
**CHEK2 Mutation**			
Present	0 (0.0%)	1 (1.1%)	1
Absent	15 (100.0%)	90 (98.9%)	
**PTEN Mutation**			
Present	0 (0.0%)	0 (0.0%)	1
Absent	15 (100.0%)	91 (100.0%)	

**ATR Mutation**			
Present	1 (6.7%)	2 (2.2%)	0.3703
Absent	14 (93.3%)	89 (97.8%)	
**BBC3 Mutation**			
Present	0 (0.0%)	0 (0.0%)	1
Absent	15 (100.0%)	91 (100.0%)	

Table S4. Comparison of Early-Onset PDAC Patients Versus Late-Onset PDAC Patients Not Treated with Gemcitabine.

**TP53 Pathway**			
**Gene**	**Early-Onset Not Treated with Gemcitabine n (%)**	**Late-Onset Not Treated with Gemcitabine n (%)**	**p-value**
**TP53 Mutation**			
Present	2 (40.0%)	44 (60.3%)	0.396
Absent	3 (60.0%)	29 (39.7%)	
**MDM2 Mutation**			
Present	0 (0.0%)	1 (1.4%)	1
Absent	5 (100.0%)	72 (98.6%)	
**MDM4 Mutation**			
Present	0 (0.0%)	0 (0.0%)	1
Absent	5 (100.0%)	73 (100.0%)	
**CDKN1A Mutation**			
Present	0 (0.0%)	0 (0.0%)	1
Absent	5 (100.0%)	73 (100.0%)	
**CDKN2A Mutation**			
Present	1 (20.0%)	14 (19.2%)	1
Absent	4 (80.0%)	59 (80.8%)	
**ATM Mutation**			
Present	0 (0.0%)	3 (4.1%)	1
Absent	5 (100.0%)	70 (95.9%)	
**CHEK1 Mutation**			
Present	0 (0.0%)	0 (0.0%)	1
Absent	5 (100.0%)	73 (100.0%)	
**CHEK2 Mutation**			
Present	0 (0.0%)	0 (0.0%)	1
Absent	5 (100.0%)	73 (100.0%)	
**PTEN Mutation**			

Present	0 (0.0%)	0 (0.0%)	1
Absent	5 (100.0%)	73 (100.0%)	
**ATR Mutation**			
Present	0 (0.0%)	1 (1.4%)	1
Absent	5 (100.0%)	72 (98.6%)	
**BBC3 Mutation**			
Present	0 (0.0%)	0 (0.0%)	1
Absent	5 (100.0%)	73 (100.0%)	

Table S5. Comparison of Early-Onset PDAC Patients Treated with Gemcitabine Versus Those Not Treated with Gemcitabine.

**PI3K Pathway**			
**Gene**	**Early-Onset Treated with Gemcitabine n (%)**	**Early-Onset Not Treated with Gemcitabine n (%)**	**p-value**
**PTEN Mutation**			
Present	0 (0.0%)	0 (0.0%)	1
Absent	15 (100.0%)	5 (100.0%)	
**PIK3R1 Mutation**			
Present	0 (0.0%)	0 (0.0%)	1
Absent	15 (100.0%)	5 (100.0%)	
**PIK3R2 Mutation**			
Present	0 (0.0%)	0 (0.0%)	1
Absent	15 (100.0%)	5 (100.0%)	
**PIK3R3 Mutation**			
Present	0 (0.0%)	0 (0.0%)	1
Absent	15 (100.0%)	5 (100.0%)	
**PIK3CA Mutation**			
Present	1 (6.7%)	0 (0.0%)	1
Absent	14 (93.3%)	5 (100.0%)	
**INPP4B Mutation**			
Present	0 (0.0%)	0 (0.0%)	1
Absent	15 (100.0%)	5 (100.0%)	
**AKT1 Mutation**			
Present	0 (0.0%)	0 (0.0%)	1
Absent	15 (100.0%)	5 (100.0%)	

**AKT2 Mutation**			
Present	0 (0.0%)	0 (0.0%)	1
Absent	15 (100.0%)	5 (100.0%)	
**AKT3 Mutation**			
Present	0 (0.0%)	0 (0.0%)	1
Absent	15 (100.0%)	5 (100.0%)	
**PPP2R1A Mutation**			
Present	0 (0.0%)	0 (0.0%)	1
Absent	15 (100.0%)	5 (100.0%)	
**TSC1 Mutation**			
Present	0 (0.0%)	0 (0.0%)	1
Absent	15 (100.0%)	5 (100.0%)	
**TSC2 Mutation**			
Present	0 (0.0%)	0 (0.0%)	1
Absent	15 (100.0%)	5 (100.0%)	
**STK11 Mutation**			
Present	0 (0.0%)	0 (0.0%)	1
Absent	15 (100.0%)	5 (100.0%)	
**RHEB Mutation**			
Present	0 (0.0%)	0 (0.0%)	1
Absent	15 (100.0%)	5 (100.0%)	
**RICTOR Mutation**			
Present	0 (0.0%)	0 (0.0%)	1
Absent	15 (100.0%)	5 (100.0%)	
**MTOR Mutation**			
Present	0 (0.0%)	0 (0.0%)	1
Absent	15 (100.0%)	5 (100.0%)	
**RPTOR Mutation**			
Present	1 (6.7%)	0 (0.0%)	1
Absent	14 (93.3%)	5 (100.0%)	

Table S6. Comparison of Late-Onset PDAC Patients Treated with Gemcitabine Versus Those Not Treated with Gemcitabine.

**PI3K Pathway**

Gene	Late-Onset<br>Treated with Gemcitabine<br>n (%)	Late-Onset<br>Not Treated with Gemcitabine<br>n (%)	p-value
**PTEN Mutation**			
Present	0 (0.0%)	0 (0.0%)	1
Absent	91 (100.0%)	73 (100.0%)	
**PIK3R1 Mutation**			
Present	1 (1.1%)	0 (0.0%)	1
Absent	90 (98.9%)	73 (100.0%)	
**PIK3R2 Mutation**			
Present	1 (1.1%)	0 (0.0%)	1
Absent	90 (98.9%)	73 (100.0%)	
**PIK3R3 Mutation**			
Present	1 (1.1%)	0 (0.0%)	1
Absent	90 (98.9%)	73 (100.0%)	
**PIK3CA Mutation**			
Present	4 (4.4%)	0 (0.0%)	0.1295
Absent	87 (95.6%)	73 (100.0%)	
**INPP4B Mutation**			
Present	1 (1.1%)	0 (0.0%)	1
Absent	90 (98.9%)	73 (100.0%)	
**AKT1 Mutation**			
Present	0 (0.0%)	0 (0.0%)	1
Absent	91 (100.0%)	73 (100.0%)	
**AKT2 Mutation**			
Present	1 (1.1%)	0 (0.0%)	1
Absent	90 (98.9%)	73 (100.0%)	
**AKT3 Mutation**			
Present	1 (1.1%)	0 (0.0%)	1
Absent	90 (98.9%)	73 (100.0%)	
**PPP2R1A Mutation**			
Present	2 (2.2%)	0 (0.0%)	0.503
Absent	89 (97.8%)	73 (100.0%)	
**TSC1 Mutation**			
Present	1 (1.1%)	0 (0.0%)	1
Absent	90 (98.9%)	73 (100.0%)	
**TSC2 Mutation**			

Present	2 (2.2%)	0 (0.0%)	0.503
Absent	89 (97.8%)	73 (100.0%)	
**STK11 Mutation**			
Present	2 (2.2%)	2 (2.7%)	1
Absent	89 (97.8%)	71 (97.3%)	
**RHEB Mutation**			
Present	0 (0.0%)	0 (0.0%)	1
Absent	91 (100.0%)	73 (100.0%)	
**RICTOR Mutation**			
Present	2 (2.2%)	0 (0.0%)	0.503
Absent	89 (97.8%)	73 (100.0%)	
**MTOR Mutation**			
Present	3 (3.3%)	0 (0.0%)	0.2545
Absent	88 (96.7%)	73 (100.0%)	
**RPTOR Mutation**			
Present	1 (1.1%)	0 (0.0%)	1
Absent	90 (98.9%)	73 (100.0%)	

Table S7. Comparison of Early-Onset PDAC Patients Versus Late-Onset PDAC Patients Treated with Gemcitabine.

**PI3K Pathway**			
**Gene**	**Early-Onset Treated with Gemcitabine n (%)**	**Late-Onset Treated with Gemcitabine n (%)**	**p-value**
**PTEN Mutation**			
Present	0 (0.0%)	0 (0.0%)	1
Absent	15 (100.0%)	91 (100.0%)	
**PIK3R1 Mutation**			
Present	0 (0.0%)	1 (1.1%)	1
Absent	15 (100.0%)	90 (98.9%)	
**PIK3R2 Mutation**			
Present	0 (0.0%)	1 (1.1%)	1
Absent	15 (100.0%)	90 (98.9%)	
**PIK3R3 Mutation**			
Present	0 (0.0%)	1 (1.1%)	1
Absent	15 (100.0%)	90 (98.9%)	
**PIK3CA Mutation**			

Present	1 (6.7%)	4 (4.4%)	0.5411
Absent	14 (93.3%)	87 (95.6%)	
**INPP4B Mutation**			
Present	0 (0.0%)	1 (1.1%)	1
Absent	15 (100.0%)	90 (98.9%)	
**AKT1 Mutation**			
Present	0 (0.0%)	0 (0.0%)	1
Absent	15 (100.0%)	91 (100.0%)	
**AKT2 Mutation**			
Present	0 (0.0%)	1 (1.1%)	1
Absent	15 (100.0%)	90 (98.9%)	
**AKT3 Mutation**			
Present	0 (0.0%)	1 (1.1%)	1
Absent	15 (100.0%)	90 (98.9%)	
**PPP2R1A Mutation**			
Present	0 (0.0%)	2 (2.2%)	1
Absent	15 (100.0%)	89 (97.8%)	
**TSC1 Mutation**			
Present	0 (0.0%)	1 (1.1%)	1
Absent	15 (100.0%)	90 (98.9%)	
**TSC2 Mutation**			
Present	0 (0.0%)	2 (2.2%)	1
Absent	15 (100.0%)	89 (97.8%)	
**STK11 Mutation**			
Present	0 (0.0%)	2 (2.2%)	1
Absent	15 (100.0%)	89 (97.8%)	
**RHEB Mutation**			
Present	0 (0.0%)	0 (0.0%)	1
Absent	15 (100.0%)	91 (100.0%)	
**RICTOR Mutation**			
Present	0 (0.0%)	2 (2.2%)	1
Absent	15 (100.0%)	89 (97.8%)	
**MTOR Mutation**			
Present	0 (0.0%)	3 (3.3%)	1
Absent	15 (100.0%)	88 (96.7%)	
**RPTOR Mutation**			
Present	1 (6.7%)	1 (1.1%)	0.2642
Absent	14 (93.3%)	90 (98.9%)	

Table S8. Comparison of Early-Onset PDAC Patients Versus Late-Onset PDAC Patients Not Treated with Gemcitabine.

PI3K Pathway			
**Gene**	**Early-Onset Not Treated with Gemcitabine n (%)**	**Late-Onset Not Treated with Gemcitabine n (%)**	**p-value**
**PTEN Mutation**			
Present	0 (0.0%)	0 (0.0%)	1
Absent	5 (100.0%)	73 (100.0%)	
**PIK3R1 Mutation**			
Present	0 (0.0%)	0 (0.0%)	1
Absent	5 (100.0%)	73 (100.0%)	
**PIK3R2 Mutation**			
Present	0 (0.0%)	0 (0.0%)	1
Absent	5 (100.0%)	73 (100.0%)	
**PIK3R3 Mutation**			
Present	0 (0.0%)	0 (0.0%)	1
Absent	5 (100.0%)	73 (100.0%)	
**PIK3CA Mutation**			
Present	0 (0.0%)	0 (0.0%)	1
Absent	5 (100.0%)	73 (100.0%)	
**INPP4B Mutation**			
Present	0 (0.0%)	0 (0.0%)	1
Absent	5 (100.0%)	73 (100.0%)	
**AKT1 Mutation**			
Present	0 (0.0%)	0 (0.0%)	1
Absent	5 (100.0%)	73 (100.0%)	
**AKT2 Mutation**			
Present	0 (0.0%)	0 (0.0%)	1
Absent	5 (100.0%)	73 (100.0%)	
**AKT3 Mutation**			
Present	0 (0.0%)	0 (0.0%)	1
Absent	5 (100.0%)	73 (100.0%)	
**PPP2R1A Mutation**			
Present	0 (0.0%)	0 (0.0%)	1
Absent	5 (100.0%)	73 (100.0%)	
**TSC1 Mutation**			

Present	0 (0.0%)	0 (0.0%)	1
Absent	5 (100.0%)	73 (100.0%)	
**TSC2 Mutation**			
Present	0 (0.0%)	0 (0.0%)	1
Absent	5 (100.0%)	73 (100.0%)	
**STK11 Mutation**			
Present	0 (0.0%)	2 (2.7%)	1
Absent	5 (100.0%)	71 (97.3%)	
**RHEB Mutation**			
Present	0 (0.0%)	0 (0.0%)	1
Absent	5 (100.0%)	73 (100.0%)	
**RICTOR Mutation**			
Present	0 (0.0%)	0 (0.0%)	1
Absent	5 (100.0%)	73 (100.0%)	
**MTOR Mutation**			
Present	0 (0.0%)	0 (0.0%)	1
Absent	5 (100.0%)	73 (100.0%)	
**RPTOR Mutation**			
Present	0 (0.0%)	0 (0.0%)	1
Absent	5 (100.0%)	73 (100.0%)	

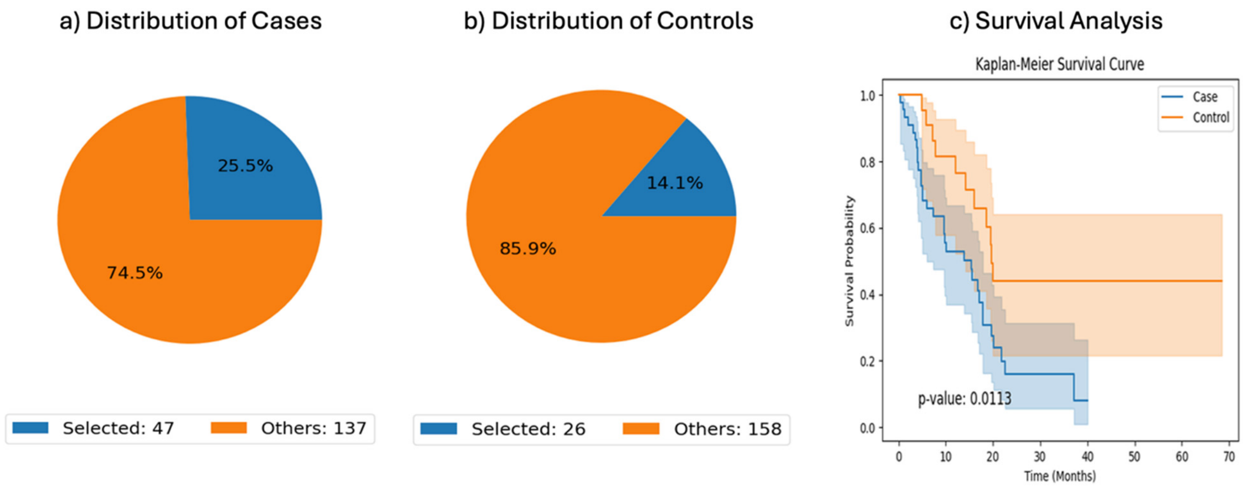

Figure S1. AI-guided cohort selection and overall survival comparison in late-onset PDAC not exposed to gemcitabine, stratified by TP53 pathway status. This supplementary figure shows how the conversational AI framework was used to define clinically specific cohorts and perform survival analysis in late-onset pancreatic ductal adenocarcinoma (PDAC) patients who did not receive gemcitabine. Based on natural language cohort criteria, the AI-HOPE-TP53 agent identified (a) a case group consisting of late-onset, non-gemcitabine-treated tumors with TP53 pathway alterations (n = 47; 25.5% of the full dataset) and (b) a control group composed of patients from the same clinical context whose tumors lacked TP53 pathway alterations (n = 26; 14.1% of the dataset). The pie charts summarize the proportion of selected and non-selected samples relative to the entire cohort. (c) Kaplan-Meier analysis demonstrated a significant difference in overall survival between these groups (log-rank p = 0.0113), with TP53 pathway-altered tumors associated with inferior survival compared with TP53 pathway-unaltered tumors. Shaded regions indicate 95% confidence intervals. These results support the prognostic relevance of TP53 pathway status in late-onset PDAC outside the setting of gemcitabine treatment and illustrate the ability of conversational AI to reproducibly generate clinically meaningful comparison cohorts.

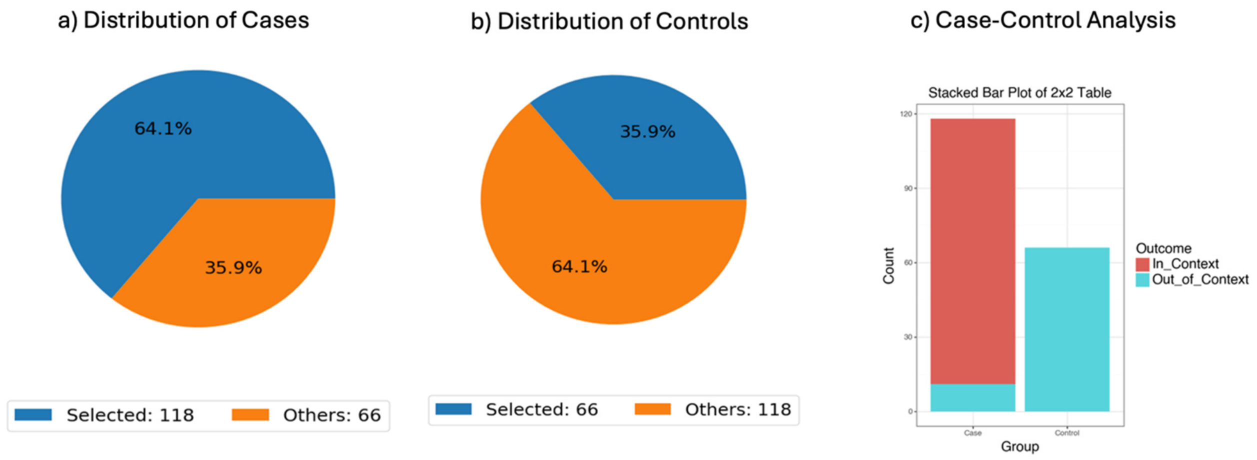

Figure S2. Conversational AI-driven comparison of TP53 pathway status within TP53-mutant PDAC tumors. This figure presents an AI-enabled enrichment analysis examining whether additional TP53 pathway alterations are differentially represented among pancreatic cancer patients whose tumors already harbor TP53 mutations. Using structured natural language criteria, the AI-HOPE-TP53 framework defined (a) a case cohort of TP53-mutant tumors with concurrent TP53 pathway alterations (n = 118; 64.1% of the dataset) and (b) a control cohort of TP53-mutant tumors lacking broader pathway-level alteration (n = 66; 35.9%). Pie charts illustrate the proportional distribution of selected versus non-selected samples across the full cohort. (c) A stacked bar plot summarizes the distribution of "in-context" (TP53 pathway-altered) and "out-of-context" (non-altered) samples across case and control groups. The majority of TP53-mutant tumors fall within the pathway-altered category, indicating substantial overlap between TP53 mutation and pathway-level dysregulation. Statistical comparison using Fisher's exact test demonstrates a highly significant association ($p \approx 0$), with an elevated odds ratio reflecting strong enrichment of TP53 pathway alterations among TP53-mutant cases. These findings reinforce the central role of TP53 as the dominant driver within its pathway and highlight how conversational AI can efficiently quantify relationships between gene-level mutations and broader pathway activation states in PDAC.

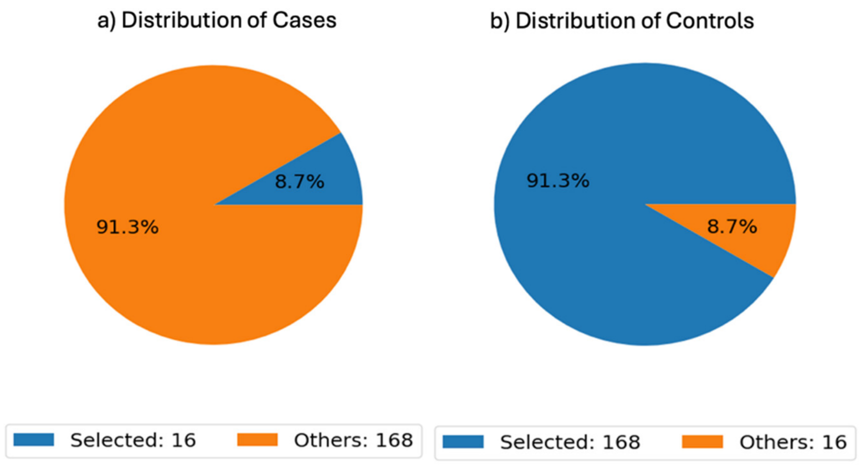

c) Association Analysis

Attribute	Test	Statistic	P-value
PI3K_Pathway	Chi-square		3.28E-39
PIK3CA	Chi-square		6.09E-11
STK11	Chi-square		1.56E-08
MTOR	Chi-square		3.73E-06
PPP2R1A	Chi-square		0.00081969
RICTOR	Chi-square		0.00081969
NLK	Chi-square		0.00081969
RPTOR	Chi-square		0.00081969
CACNA1E	Chi-square		0.00081969
TCF7	Chi-square		0.00081969
TSC2	Chi-square		0.00081969
RNF43	Chi-square		0.0050027
TCF7L1	Chi-square		0.01046913
CACNA1I	Chi-square		0.01046913
BMP5	Chi-square		0.01046913
NFATC2	Chi-square		0.01046913
WNT_Pathway	Chi-square		0.01497819
International Classification of Diseases for Oncology, Third Edition ICD-O-3 Histology Code	Chi-square		0.01504108
Gemcitabine_Treatment_Received	Chi-square		0.02336831
Gemcitabine_Treatment	Chi-square		0.02336831
Progression Free Status	Chi-square		0.03224802
APC	Chi-square		0.03872365
CACNA1G	Chi-square		0.03872365

Figure S3. Conversational AI-based identification of clinical and molecular features associated with PI3K pathway alterations in PDAC. This figure summarizes an AI-guided comparative analysis of pancreatic ductal adenocarcinoma (PDAC) tumors stratified by PI3K pathway status across the full cohort. The case cohort comprised tumors with PI3K pathway alterations (n = 16; 8.7%), while the control cohort included tumors without detectable PI3K pathway alterations (n = 168; 91.3%). Panels (a) and (b) illustrate the relative proportion of selected and non-selected samples within each group, highlighting the comparatively low prevalence of PI3K pathway-altered tumors in this dataset. Panel (c) presents the results of a comprehensive association analysis using Chi-square testing to evaluate relationships between PI3K pathway status and a wide range of clinical and genomic attributes. Significant associations were observed with multiple PI3K pathway components and related signaling genes, including PIK3CA, STK11, MTOR, PPP2R1A, RICTOR, TSC2, and RPTOR, reflecting coordinated involvement of upstream regulators and mTOR complex-associated elements. Additional associations were identified with signaling and regulatory genes such as NLK, RNF43, TCF7, and members of the CACNA family, as well as broader pathway-level features including WNT pathway alterations. Clinically, PI3K pathway status was associated with treatment-related variables, including gemcitabine exposure, and with progression-free status, suggesting a potential link between PI3K pathway activation and disease behavior. Histologic classification (ICD-O-3) also demonstrated a significant association, indicating possible variation in PI3K pathway involvement across tumor subtypes. Overall, these findings indicate that PI3K pathway-altered PDAC represents a distinct, though relatively infrequent, molecular subset characterized by coordinated alterations across multiple signaling nodes and associations with treatment and clinical outcomes. This analysis highlights the capability of conversational artificial intelligence to efficiently uncover multidimensional relationships between pathway alterations and clinicogenomic features in complex cancer datasets.

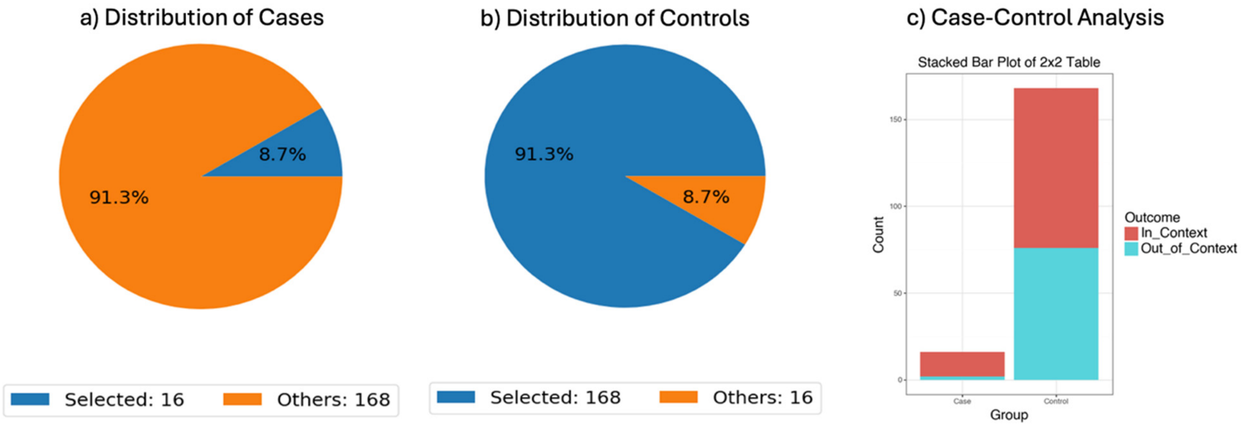

Figure S4. Conversational AI-based evaluation of gemcitabine exposure among PI3K pathway, defined PDAC subgroups. This figure illustrates an AI-driven odds ratio analysis assessing whether gemcitabine treatment is differentially represented between tumors with and without PI3K pathway alterations. Using structured query criteria within the AI-HOPE-PI3K framework, the case cohort included PI3K pathway-altered tumors (n = 16), while the control cohort consisted of PI3K pathway-unaltered tumors (n = 168). Pie charts in panels (a) and (b) depict the proportion of selected samples relative to the full dataset, highlighting the relatively small fraction of PI3K-altered cases. Panel (c) presents a stacked bar visualization comparing "in-context" (gemcitabine-treated) and "out-of-context" (non-treated) samples across both groups. Among PI3K-altered tumors, 87.5% were associated with gemcitabine exposure, compared with 54.76% in the PI3K-wild-type group. Statistical analysis demonstrated a significant difference between cohorts (Chi-square p = 0.023), with an odds ratio of 5.783 (95% CI: 1.274-26.24), indicating that PI3K pathway alterations are significantly enriched among gemcitabine-treated patients. These findings suggest a potential association between PI3K pathway activation and treatment exposure in PDAC, supporting the hypothesis that PI3K signaling may be linked to therapy-related tumor biology. More broadly, this analysis highlights the utility of conversational artificial intelligence for rapidly identifying treatment-associated molecular patterns within clinically stratified cancer datasets.
